# Supplementary material for: Interplay of structured and random interactions in complex ecosystems dynamics
Source: PLoS Comput Biol. 2025 Dec 26;21(12):e1013786. doi: 10.1371/journal.pcbi.1013786 (PMC12755798; doi:10.1371/journal.pcbi.1013786)
Supplement: Attachment — Submitted filename: Report PLOS00663.pdf [file pcbi.1013786.s002.pdf]

Referee Report for PCOMPBIOL-D-25-00663

In this work, the authors study the generalized Lotka-Volterra Equations (gLVE), a well-established theoretical model used to describe the dynamics of species abundances in ecosystems. They consider the case where the interaction matrix consists of two components: a random, structureless part with Gaussian statistics, and a low-rank deterministic component representing structured interactions. The analysis focuses on the limit of a large number of species, with the rank of the deterministic matrix held fixed. This system is amenable to the methods of disordered systems, in particular dynamical mean-field theory (DMFT). DMFT enables a statistical description of typical dynamical trajectories—"typical" with respect to realizations of the random interactions—through a set of effective stochastic equations. The coupling between species abundances is encoded in the properties of the noise of these stochastic equations, which must be determined self-consistently. After discussing this formalism, in Sec. 3.2 the authors discuss the properties of the equilibrium configuration characterizing the long-time limit of the dynamics (assuming it exists); in particular, they examine how the abundance distribution of species is shaped by the presence of both structure and randomness in the interactions. In Section 3.3, they present numerical simulations of the gLVE, now reinterpreting functional groups as species and microscopic variables as strains. They argue that as the relative strength of the random component increases, the system undergoes two transitions: first, randomness disrupts synchronization among strain trajectories within the same species, damping oscillations and driving the system to a fixed point. At higher values of randomness, this fixed point becomes unstable, reproducing a transition well-understood in the "fully unstructured" (only random interactions) model.

Overall, I believe this work merits publication and that PLOS Computational Biology is an appropriate venue. However, I also think that some sections would benefit from further clarification to improve accessibility and better connect with the existing literature. Below, I report a few questions and comments on the main text and appendices, respectively.

MAIN

1. The generalized Lotka-Volterra equations (gLVE), in the absence of a deterministic component in the interaction matrix—or in the presence of a simple rank-1 term generating a uniform average—have been extensively studied in the disordered systems literature; approaches based on dynamical mean-field theory (DMFT) and the cavity method have been used to characterize the unique equilibrium phase in such models. The authors frequently cite Ref. [3] in this context. I would suggest adding the seminal reference by Opper and Diederich, "Phase Transition and 1/f Noise in a Game Dynamical Model", which to my knowledge contains the first derivation of the "cavity" equations characterizing the unique equilibrium.

2. In Eq. (2), it is assumed that the community-level functions depend linearly on species abundances. While I understand this is a natural simplification, I wonder whether this assumption can be motivated in ore detail. The authors later mention that incorporating nonlinearities would be straightforward. If the resulting equations (such as Eq. (6)) would change in a relatively compact and interpretable way, I would encourage the authors to briefly comment on how the formulation would be modified in the nonlinear case.

3. In Section 3.1, the DMFT equations (Eq. 6) are introduced. From reading the appendix, it becomes clear that the derivation relies on the assumption that the structured part of the interaction matrix has finite rank, while the number of species tends to infinity. I would recommend making this assumption explicit in the main text as well.

4. By analogy with Eq. (7), should Eq. (8) include an explicit species index?

5. I could not find the definition of u_i^{(\mu)} in Eq. (12). Perhaps I missed it; if so, I would suggest recalling it near the equation for clarity. I have the impression it should be \mathcal{S}.

6. In Section 3.3, the authors discuss simulation results of the dynamical equations. The exact structure of the deterministic part of the interaction matrix used in these simulations is not entirely clear to me from the text: I think it would be helpful to explain more precisely how this setup relates to the general framework introduced earlier in the paper. The caption to Fig. 6 states that the interaction matrix is chosen as in [36]; to make the presentation more self-contained, I recommend briefly clarifying what this choice entails. Additionally, the simulations in Section 3.3 involve a relatively large number of "species," only about one order of magnitude smaller than the total number of strains. Should one expect that the interesting dynamics observed at small σ are still captured in the mean-field limit (finite rank, infinite S) discussed in the earlier sections?

7. Regarding the dynamics at smaller σ, the authors mention two mechanisms for destabilizing the unique stable equilibrium: one driven by extinctions caused by randomness, which suppress oscillations, and another associated with outliers in the Jacobian. It is not entirely clear to me how these two mechanisms correspond to the two features highlighted in the discussion of Fig. 6: (i) the breakdown of synchronization in strain trajectories, and (ii) the suppression of chaos and convergence to a unique stable equilibrium. In particular, the convergence to equilibrium is governed by the outlier transition?

8. The dynamical regime shown in Fig. 6(c) should, in principle, be captured by the equilibrium equations derived in Section 3.2. Given the simulation data, could the authors verify this by performing a numerical consistency check?

9. In the work arXiv:2503.20908 by Fournier et al, the chaotic dynamics of a model with a deterministic component in the interaction matrix is studied by DMFT. There appears to be a transitions within the chaotic phase, from a regime where trajectories explore the entire accessible configuration space to one where the chaotic attractor is constrained in size by the deterministic term. This seems, at least superficially, similar to the behavior observed at small σ in this work. I was wondering whether the authors see a connection, given their intuition on the features of the dynamics at small σ.

APPENDICES

1. In Section D, could the authors clarify the justification for neglecting extinctions and for dividing the dynamical equation by x_i(t) inside the delta functional in Eq. (S11)?

2. In Eq. (S22), should m(t) be f(t)? Also, the index lambda seems to be missing, as in (S23)?

3. Regarding Eqs. (S24)–(S27): \mathbb{P}(\vec x) is a function of the dynamical variables x_i, and after introducing (S20), there is still a term depending explicitly on the x_i, in addition to the terms \psi_i: this is the term containing \mathcal{I}_i^{(\lambda)}. I have the impression that this term does not appear correctly in (S27-S28), as it appears multiplied by \hat{x}_i and not {x}_i. For the same reason, Eq. (S37) also seems incorrect to me.

4. Regarding Eq. (S38): in the "standard" DMFT, derivation the correlation function between the hatted variables is averaged over both trajectories x_i (t) and \hat{x}^_i (t), which are correlated. It seems to me that averaging over x_i (t) is necessary to justify the vanishing of this term. Could the authors explain why this is not required here, and why the correlation in Eq. (S38) vanishes? I also missed why this vanishing implies that the average of \hat{n}_\lambda(t) is zero —perhaps this is why this equation is not clear to me.

5. Unless I am mistaken, the results of Appendix G are not referenced in the main text.

TYPOS:

- On line 289, it is stated that bulk modes "diverge," though I believe the intended meaning is that they vanish.
- just below Eq. (9), the sentence beginning with "for convenience" is probably a typo.

- Eq. (S13): the dot
- Eq. (S14): the d_{zij}
- In (S33) and (S34), the index in the average should be j I think
- “Calculationn” on line 586, “eignevalues” before eq. (19)
