# Supplementary material for: Interplay of structured and random interactions in complex ecosystems dynamics
Source: PLoS Comput Biol. 2025 Dec 26;21(12):e1013786. doi: 10.1371/journal.pcbi.1013786 (PMC12755798; doi:10.1371/journal.pcbi.1013786)
Supplement: Attachment — Submitted filename: PLOS Comments.pdf [file pcbi.1013786.s004.pdf]

Editor

All three reviewers agree that your manuscript has the potential to make a significant contribution as it bridges classic disordered-interaction models to function-based ecological structure, and it showcases how DMFT can be extended to this hybrid setting. At the same time, they all raise concerns about clarity of presentation. In addition, both Reviewer 1 and Reviewer 3 point out that the manuscript needs to be better situated within the existing literature to clarify its novel contributions.

Dear Editor,
Thank you very much for allowing us to resubmit this manuscript, and for the many helpful comments that have allowed us to notably rework, and hopefully improve, the general presentation of our manuscript and its results.

I find myself in agreement that the presentation, including the figures as noted by Reviewer 2, undersell your results. For example, outside of the plankton model, it is never entirely clear what the coarse-grained quantities are—e.g. if total biomass of functional groups, then what defines a functional group? I also found the discussion to be quite technical and likely to be understood and appreciated only by a very specialized audience. In your revision, you should translate your mathematical results and their significance to a broader audience, more clearly identifying which aspects of the community are highly sensitive to hidden variation and which appear broadly robust, and spell out what this means for noise-driven changes in stability and dynamics.

In order to help the reader follow our narrative, and according to the comments of the Reviewers, we have changed all the figures so that they are explicitly linked to one another and use the same notations introduced by the first two illustrative schemes. The new Figure 2 summarizes the relevant theoretical predictions of models with purely random or purely structured (according to our definition, now illustrated schematically in Figure 1) interactions. There, we also introduce the notations that are used in following figures, notably the distinction between microscopic and macroscopic variables and the community-level functions. We have rearranged the following figures and moved some of the old as supplementary figures. Theoretical predictions of the interplay of randomness and structure on the SAD, macroscopic functions, and dynamics are now worked out in detail for the intuitive example of functional groups.

We have significantly revised the Discussion so as to make it less technical, and to identify more clearly what community-level features are sensitive or robust to the presence of disorder.

L170. What are the coarse-grained variables?

We have reformulated this paragraph following suggestions from the referees.

P8 Footnote: what are the u’s and v’s?

We apologize for this typo, due to a different notation in an earlier version of the manuscript. These are in fact the I’s and S’s. We have changed it.

Figure 2: If I understand this figure correctly, I believe the black blocks should be on entries (1,3), (2,1), (3,2) rather than on (1,2), (2,3), (3,1). Also, the figure needs to better explain that the dark segments represent sensibility (sensitivity?) and impact of species *i* with respect to group lambda, which is not at all clear.

Following suggestions by the reviewers, we have removed this figure entirely to fuse former figures 1 and 2, hoping to make them more immediately clear and remove finer details that are unneeded at that stage in the article. We have moved one of the former figures as a supplementary figure for further illustration of certain special cases where our formalism apply.

L207. These effect traits are never explained. Are they the same as calligraphic S?

This is the same typo as in P8 Footnote.

L234. It is not at all clear from Eq. 15 that this represents the survival probability.

We have amended this to explain the definition of the survival probability.

L241. This needs a more detailed explanation.

We have rewritten the paragraph to clarify the phenomenon illustrated in Fig. 4.

Fig. 4 caption: (d) proportion, not number, of surviving species.

This has been rewritten and we now avoid referring to numbers.

L243. Wouldn’t the observed diversity in your simulations depend on the abundance cutoff used to determine which species survive?

In the presence of migration, species that would go extinct at the fixed point quickly reach abundances close to the migration threshold. On the other hand, the minimal non-zero abundance in a community with S species will be of order $1/S$. Thus, as long as the migration threshold is chosen sufficiently small (in our case, $m=10^{-8}$ which is much smaller than $1/S$ for the values of S that we use), the set of extinct species and the set of extant species are typically well separated. We have specified the value of m after its definition.

Reviewer 1

In this work, the authors study the generalized Lotka-Volterra Equations (gLVE), a well-established theoretical model used to describe the dynamics of species abundances in ecosystems. They consider the case where the interaction matrix consists of two components: a random, structureless part with Gaussian statistics, and a low-rank deterministic component representing structured interactions. The analysis focuses on the limit of a large number of species, with the rank of the deterministic matrix held fixed. This system is amenable to the methods of disordered systems, in particular dynamical mean- eld theory (DMFT). DMFT enables a statistical description of typical dynamical trajectories—“typical” with respect to realizations of the random interactions —through a set of effective stochastic equations. The coupling between species abundances is encoded in the properties of the noise of these stochastic equations, which must be determined self-consistently. After discussing this formalism, in Sec. 3.2 the authors discuss the properties of the equilibrium con guration characterizing the long-time limit of the dynamics (assuming it exists); in particular, they examine how the abundance distribution of species is shaped by the presence of both structure and randomness in the interactions. In Section 3.3, they present numerical simulations of the gLVE, now reinterpreting functional groups as species and microscopic variables as strains. They argue that as the relative strength of the random component increases, the system undergoes two transitions: first, randomness disrupts synchronization among strain trajectories within the same species, damping oscillations and driving the system to a fixed point. At higher values of randomness, this fixed point becomes unstable, reproducing a transition well-understood in the “fully unstructured” (only random interactions) model.

Overall, I believe this work merits publication and that PLOS Computational Biology is an appropriate venue. However, I also think that some sections would bene t from further

clarification to improve accessibility and better connect with the existing literature. Below, I report

a few questions and comments on the main text and appendices, respectively.

We thank the referee for their positive feedback and for taking the time to make such a detailed review of the manuscript. Please find below the answers to their recommendations.

MAIN

1. The generalized Lotka-Volterra equations (gLVE), in the absence of a deterministic component

in the interaction matrix—or in the presence of a simple rank-1 term generating a uniform

average—have been extensively studied in the disordered systems literature; approaches

based on dynamical mean-field theory (DMFT) and the cavity method have been used to

characterize the unique equilibrium phase in such models. The authors frequently cite Ref. [3]

in this context. I would suggest adding the seminal reference by Opper and Diederich, "Phase

Transition and 1/f Noise in a Game Dynamical Model", which to my knowledge contains the

first derivation of the "cavity" equations characterizing the unique equilibrium.

We thank the referee for suggesting this reference. We have added it when introducing DMFT.

2. In Eq. (2), it is assumed that the community-level functions depend linearly on species

abundances. While I understand this is a natural simplification, I wonder whether this

assumption can be motivated in more detail. The authors later mention that incorporating

nonlinearities would be straightforward. If the resulting equations (such as Eq. (6)) would

change in a relatively compact and interpretable way, I would encourage the authors to briefly

comment on how the formulation would be modified in the nonlinear case.

We now clarify that the linearity assumptions are needed to fully encapsulate functional structure in a simple interaction matrix, but that we could add nonlinearities either in the way that functions depend on species abundances (going toward higher-order interactions) or that species growth depends on functions (going toward functional responses), and cite previous works in that spirit. We expect that some such modifications would easily follow from existing work (for instance, having a linear combination of variables feed into a nonlinear functional response would extend ideas developed in refs Barbier 2018 or Sidhom et al 2020), while others would require additional work and might have much more complex consequences (we have a manuscript in preparation that interrogates this point, and shows that significantly different outcomes may arise when we lose the property of having a large sum of independent contributions for the random component).

3. In Section 3.1, the DMFT equations (Eq. 6) are introduced. From reading the appendix, it becomes clear that the derivation relies on the assumption that the structured part of the interaction matrix has finite rank, while the number of species tends to infinity. I would recommend making this assumption explicit in the main text as well.

In addition to $n_F\ll S$ being mentioned at the beginning of Sec. 2.2 (line …), we have added *“Given that $n_F \ll S$, the total interaction matrix is the superposition of a low-rank term and a random term.”* in Sec 2.3.

4. By analogy with Eq. (7), should Eq. (8) include an explicit species index?

We thank the referee for pointing out the typo in Eq. (7). The index i disappears whenever we average over species, as signalled by the overline.

5. I could not find the definition of u_i^{(\mu)} in Eq. (12). Perhaps I missed it; if so, I would suggest recalling it near the equation for clarity. I have the impression it should be \mathcal{S}.

This is indeed a notation carried over from a previous version of the manuscript, our apologies. We have replaced $u_i^{(\mu)}$ by $\mathcal{S}_i^{(\mu)}$.

In Section 3.3, the authors discuss simulation results of the dynamical equations. The exact structure of the deterministic part of the interaction matrix used in these simulations is not entirely clear to me from the text: I think it would be helpful to explain more precisely how this setup relates to the general framework introduced earlier in the paper. The caption to Fig. 6 states that the interaction matrix is chosen as in [36]; to make the presentation more self-

contained, I recommend briefly clarifying what this choice entails. Additionally, the simulations in Section 3.3 involve a relatively large number of “species,” only about one order of magnitude smaller than the total number of strains. Should one expect that the interesting dynamics observed at small σ are still captured in the mean-field limit (finite rank, infinite S) discussed in the earlier sections?

We have reworked the presentation of Section 3.3 in line with these comments and the recommendations of the other referees.

We thank the referee for raising the interesting question of the effects of finite group size. The assumption of finite rank, which reflects typical ecological knowledge of communities, allows to safely perform the saddle-point calculation in the DMFT derivation. While we have no clear statement about how far one can go for finite S, we note that due to the interactions between groups being strong, the effective number of groups present at any given time (i.e. not at the migration threshold) is much smaller than the number of groups in the pool. Since the dynamics are chiefly determined by these groups, and the effective dimensionality of the macroscopic dynamics is small, we expect the saddle calculation to keep holding.

7. Regarding the dynamics at smaller σ, the authors mention two mechanisms for destabilizing the unique stable equilibrium: one driven by extinctions caused by randomness, which suppress oscillations, and another associated with outliers in the Jacobian. It is not entirely clear to me how these two mechanisms correspond to the two features highlighted in the discussion of Fig. 6: (i) the breakdown of synchronization in strain trajectories, and (ii) the suppression of chaos and convergence to a unique stable equilibrium. In particular, the convergence to equilibrium is governed by the outlier transition?

We have reworked Fig. 6 (now Fig. 5) and added the projection on a plane to illustrate the two different kinds of stability loss. Additionally, in order to comply with the comments of another referee, we have dropped the species/strain terminology and used the groups/species one that is used throughout the rest of the paper.

There are two types of chaos in this model: synchronous chaos and asynchronous chaos. In the first, both groups and species fluctuate chaotically, and species remain synchronized with their groups. In the second, which is akin to the chaos of the “standard” rGLV, species fluctuate chaotically, but lack of synchrony means that fluctuations are suppressed at the level of the groups. Synchronous chaos in fact is qualitatively similar to that of Mallmin et al., and happens for small σ. Asynchronous chaos happens for larger σ. For intermediate σ, the system converges to a fixed point. Starting from the fixed point phase and decreasing σ leads to some outliers of the Jacobian becoming unstable: this signals the transition to synchronized dynamics (first in the form of synchronous cycles, as in Fig. 5 B), and

later synchronized chaos. On the other hand, increasing σ leads to a destabilization of the bulk and asynchronous chaos.

8. The dynamical regime shown in Fig. 6(c) should, in principle, be captured by the equilibrium equations derived in Section 3.2. Given the simulation data, could the authors verify this by performing a numerical consistency check?

We have indeed checked that the simulation data is consistent with the equilibrium condition based on $\Jcal$, and we have added a Supplementary Figure illustrating the eigenvalue spectrum for the two relevant regimes of cyclic oscillations and randomness-induced stability (panels B and C of Fig.5). The real part of the leading eigenvalues of $\Jcal$ right before and right after the transition to stability (for the critical value σ indicated in the main text) have opposite signs.

9. In the work arXiv:2503.20908 by Fournier et al, the chaotic dynamics of a model with a deterministic component in the interaction matrix is studied by DMFT. There appears to be a transitions within the chaotic phase, from a regime where trajectories explore the entire accessible configuration space to one where the chaotic attractor is constrained in size by the deterministic term. This seems, at least superficially, similar to the behavior observed at small σ in this work. I was wondering whether the authors see a connection, given their intuition on the features of the dynamics at small σ.

We thank the referee for pointing this interesting connection to us. In our model, the dynamics at sufficiently small σ is indeed constrained but only at short times, i.e. as long as the dominant groups stay the same. For long-enough times, however, there is a turnover at the level of the groups similar to that of Mallmin et al., so that the trajectory ends up visiting the whole phase space. In the work by Fournier et al., symmetry is broken at all time scales. In that sense, the two are different.

However, for increased σ the situation can become more similar to that of Fournier et al. In the second panel of Fig. 5, the dynamics becomes cyclic so that the set of abundant groups is held constant, i.e. disorder localizes the macroscopic dynamics. This localization is carried onto the fixed point phase (necessarily, since the number of groups that dominate must be small) and also, from numerical simulations, onto the phase of microscopic chaos (see for instance Fig. 5 of the revised manuscript, where the groups that dominate in the 4th row are stable and coincide with those of the 3rd row). At this stage, we cannot rule out finite-size effects or insufficient simulation time but at the very least we can say that if there is still group turnover in the phase of microscopic chaos then its time scale must be much longer than that of group turnover at small $\sigma=0$. Such “macroscopically-induced localization of microscopic chaos” undoubtedly deserves investigation in future work.

APPENDICES

1. In Section D, could the authors clarify the justification for neglecting extinctions and for dividing the dynamical equation by x_i(t) inside the delta functional in Eq. (S11)?

This is an interesting technical point. Implicit in the derivation is the assumption that the starting abundances are non-zero (in the absence of migration). This does not reduce the generality, since extinct species could be removed from the model altogether. As long as this is verified, species cannot go extinct because the gLV equations are order-one differential equations. Hence the division is safe in the path integral. Note that this doesn't contradict the existence of a fixed point where species go extinct: fixed points are a static property of the system, and they are never really reached by the dynamics.

We have added a small explanation around (S11).

2. In Eq. (S22), should m(t) be f(t)? Also, the index lambda seems to be missing, as in (S23)?

We thank the referee for such a detailed revision of our SupMat. This is indeed a typo coming from a previous version of the manuscript. We have corrected all instances of it.

3. Regarding Eqs. (S24)–(S27): \mathbb{P}(\vec x) is a function of the dynamical variables x_i, and after introducing (S20), there is still a term depending explicitly on the x_i, in addition to the terms \psi_i: this is the term containing \mathcal{I}_i^{(\lambda)}. I have the impression that this term does not appear correctly in (S27-S28), as it appears multiplied by \hat{x}_i and not {x}_i. For the same reason, Eq. (S37) also seems incorrect to me.

There was indeed a typo in both equations, which we have now amended. In (S28), the variables $\hat{f}$ are multiplied x_i, while the variables $\hat{n}$ are correct. In (S38), it is n_λ that equals 0, and therefore so does $\hat{f}\lambda$ as per the saddle equations. With these typos corrected, in (S24-27) nothing depends on i except for ψ_i.

4. Regarding Eq. (S38): in the "standard" DMFT derivation, the correlation function between the hatted variables is averaged over both trajectories x_i (t) and \hat{x}_i (t), which are correlated. It seems to me that averaging over x_i (t) is necessary to justify the vanishing of this term. Could the authors explain why this is not required here, and why the correlation in

Eq. (S38) vanishes? I also missed why this vanishing implies that the average of \hat{n}

_\lambda(t) is zero —perhaps this is why this equation is not clear to me.

We thank the referee for pointing out this subtle issue. The short answer is that the average is indeed missing an integral over non-hatted variables. However, given that this is not totally apparent from the derivation, we give below a detailed explanation.

Usually, the DMFT derivation deals with the partition (or characteristic) function of the system. Here, in order to use as little technical terminology as possible, we have made the choice of taking the probability of trajectories as the central object of the derivation.

Hence, the usual statement of the saddle method ("the integral equals the maximum value of the integrand") is replaced by a statement about concentration: "if trajectories are grouped depending on the macroscopic order parameters, then the probability of a given set of order parameters concentrates around a typical value". Crucially (and this is the missing bit that troubled the referee), this typical value is not obtained by only extremizing the probability of individual trajectories: it must also take entropy (i.e. the number of trajectories compatible with given macroscopic parameters) into account. This precisely enters as an integral over the non-hatted variables, so that what one ends up extremizing is, in fact, the partition function. Thus the derivation is coherent with what the referee expected.

We have rephrased the second half of this Section in order to make this more clear while avoiding to use terminology such as "partition function".

5. Unless I am mistaken, the results of Appendix G are not referenced in the main text.

Indeed, we have now referenced them in the main text when introducing the random part of the interactions.

TYPOS:

- On line 289, it is stated that bulk modes "diverge," though I believe the intended meaning is that they vanish.

Our phrasing was indeed vague. We have changed the sentence to "bulk eigenvalues become unstable" in order to avoid confusion between "eigenvalue" terminology (eigenvalues indeed vanish) and linear dynamical modes (which diverge when the eigenvalues vanish).

- just below Eq. (9), the sentence beginning with "for convenience" is probably a typo.-

Indeed, and we thank the referee for spotting this. We have removed the sentence.

Eq. (S13): the dot

Eq. (S14): the d_{zij}

In (S33) and (S34), the index in the average should be j I think

“Calculationn” on line 586, “eignevalues” before eq. (19)

We have corrected all these typos.

Reviewer 2

The manuscript by Giral Martinez et al applies advanced analytical techniques to address an important and timely question: to what extent do the predictions of random models (a staple of a large subfield of theoretical ecology) apply in structured communities? To address this, the authors introduce a clever model that combines structured and disordered interactions in a way that is both plausible and combines the strengths of the two dominant modeling frameworks (Lotka-Volterra, which is in some sense "very general" but purely compositional, and more "functional" frameworks like resource competition or functional guilds). They show that a powerful analytical technique (DMFT) can be productively applied in their model, and use it to draw some conclusions about the dynamics. The manuscript is an impressive contribution, and with some changes could be quite impactful. However, I do think a few changes to presentation may be required for this work to actually attain this impact.

My main concern is that the work, as written, will fail to reach the target audience. The model and the approach proposed here are striving to do something big and impactful. Today, there is a big tension: powerful stat physics methods can calculate so much, but in the context of "silly" random models that seem very far from anything real. This manuscript is an important step towards resolving this tension. The authors have made some commendable decisions, e.g. explaining the idea of DMFT in reasonably accessible way, by packaging away much of the technical detail into the supplement, and by making this supplement quite detailed and reasonably pedagogical. I applaud this effort. However, in my mind, the "punchline" figures in the main text entirely fail to communicate what the manuscript is achieving - no biologist will find them relatable or impressive (and physicists would arguably struggle with this as well…).

In summary, I believe this work is potentially very impressive, but is let down by serious issues with presentation.

Thank you very much for this positive assessment of our manuscript’s potential, and for the help with improving the presentation to get it across. Our dip into the technical side has made us feel that, back on the more conceptual side, a number of results become easier to parse using just a few intuitions that are worth laying out in the open. We think that this can speak to (somewhat theoretical) biologists as well as (somewhat ecological) physicists. We hope the current presentation does a better job at conveying these intuitions, and providing enough technique to substantiate them without obfuscating the conceptual content.

Major issues:
(1) This is by far the biggest. To me, Figs. 4-6 aren't working. It is not my role to tell the authors what the figures should rather be - they are the best judges of what constitutes the most impressive aspects of their results. However, the readership of PLOS Comp Biology is generally not made of statistical physicists, and even physicists skimming the figures wouldn't know why these results are supposed to be exciting. So I urge the authors to recruit a biologist friend and ask them for feedback on these figures, especially 4 and 5. I am quite certain that they massively undersell the actual results.

We agree and we have reworked the figures to clarify what they contribute to the overall message. We have merged figures 1 and 2, added a new Figure 2 that captures what are the basic predictions from pure structure or pure randomness, whose interplay we will study. The remaining figures (3, 4, and 5, the latter being former figure 6) each highlight something simple and something surprising about one of these basic predictions, with consistent notations. We hope that, through the structuring role of Figures 1 and 2 and some revisions, the contributions of the others become much clearer.

Here are some things that might help.
(1a) Consider replacing figure titles by a statement of what one is meant to conclude, not what is plotted.
Let's take Fig. 6. Current title: "Influence of strain variability on species and strain dynamics". In reality, the figure shows that randomness can stabilize oscillating communities, but in a very interesting non-monotonic way, with a disorder-induced transition. This is fascinating, and would be completely lost on the reader based on the title. Thankfully, here the figure is visual enough that this may still come across. But Fig. 4 & 5 are not so fortunate:

- "Fig 4: Species abundance distributions and community-level observables at equilibrium" - what about these SADs should the reader find interesting? That they are well predicted by the model? That the structured interactions changes them dramatically? Doesn't change them at all? That there are multiple regimes? What is the biologically relevant statement? Etc. etc. Fig. 4a and 4b almost seem like they are illustrating a minor technical point. Is this the key result that deserves being showcased in the prime real estate of the punchline figure Fig. 4? What feature of Fig. 4cd are we meant to be impressed by? That these curves increase, decrease? That the lines pass through the dots? that panels c and d are different?
- "Fig 5: Species abundance distributions for communities with a power-law distributed structural trait." Why are we interested in a power-law distributed structural trait? This title makes the figure seem like some minor technical detail.

Some of these points are explained much better in the text. BUT people look at the figures to decide whether to read the text. Poorly designed figures will obliterate the impact of the paper. And furthermore, crystallizing out the impact that would speak to biologists would improve the text as well (even for physics-trained readers).

Thank you, these are very interesting suggestions. We have tried to improve figure captions by retaining a shorter title than what we would write if we were to fully state its conclusions. We detail immediately after the main message, before entering into finer details, in the hope that

even a cursory reading of the figures will let the main ideas shine through. We have moved former Fig 5 to the supp mat as it was making a second-order point.

(1b) The premise of the paper is to investigate how predictions or behaviors change when one tun3s the relative impact of structure vs random interactions. This, I believe, is the entire point & strength of the model - to provide a knob one could turn to interpolate between "fully random" and "highly structured". But then, the punchline plots has to be "something against sigma": the two extremes that are well understood, the middle that is new, exciting, and interesting. In the current exposition, the only such plot is the very last panel of the very last figure.

Now hopefully the “something against sigma” is clearer in figures 3 to 5.

(2) Continuing with the presentation issues: I found Figs. 1 & 2 hard to parse.
• In Fig. 1 I'd recommend making the arrowheads larger to be more visible.
• Legend refers to lambda, but there is no lambda in the figure. Is lambda labeling the box (green/blue)?
• In both figures, I think readability could be greatly enhanced by a strategic placement of labels ("species; functions; effect traits, sensitivity traits") that would make notations understandable without constantly referring to the figure legend. In Fig. 1, it might be helpful to explicitly label the two sides along the lines of "interactions structured by functional groups" and "resource-mediated interactions".
• In Fig. 1a, it looks like the two black arrows are jointly labeled by S^(1)_j. Perhaps labeling the other arrow as S^(1)_i would make this figure easier to understand. (Fig. 1b is better in this sense, with all three arrows labeled correctly).
• A separate legend for i and j being two *green* shapes in confusing. The existence of same shapes in both green and blue box is also confusing. Is this intentional? Are the blue and green dots the same species (that can contribute to two groups), or are they different species?
• To non-programmers, starting the numbering from zero is confusing. It seems to imply that 0 is somehow special. Besides, Fig. 1 numbers the two groups as 1,2 and not 0,1.
• In Fig. 2 I can understand the "plus" between (a,b) and (c,d), but the plus between species is confusing as it suggests the vectors are added, which they are not.

We have removed these two figures, which seemed to confuse more than they clarified, from the main text (one is in the Supplementary Information), and substituted them with a single, simpler one with fewer unnecessary details, i.e. the new Figure 1. We tried to attend to all your suggestions in doing so.

(3) This is more of a "medium" issue, and has to do with contextualization of the study.

Our goal here is to investigate which predictions of fully random models are altered by structure in species interactions. Among possible predictions, only equilibrium stability has received extensive attention along this line so far.

I agree, but I'm not sure this passage is entirely fair. The value of the question is recognized

rather broadly. It's true that within gLV-based work, an outsized fraction has focused on equilibrium stability, but gLV simply doesn't offer very many options in terms of relevant observables, so diversity and stability reign supreme. But investigating the extent to which random-model predictions survive relevant levels of structure (including how this interacts with specifically the ability to coarse-grain ecosystems) is already recognized to be broader. Some theorists interested in similar questions choose to use resource competition models (Ben Good, Mikhail Tikhonov) -- precisely because that framework allows accessing other (more "functional") properties. There's also the question of the extent to which large-scale model predictions depend on other interaction-structuring "details", such as metabolism (Avi Flamholz, Daniel Segre, Terry Hwa) or resource preference hierarchies (Sergei Maslov, Akshit Goyal).

Obviously, there's no need for the authors to review *all* that work here, but mentioning some of this would actually help underscore the cleverness of the authors' approach as it actually marries gLV with elements of those "functional" models. It would also make their work accessible & appealing to a broader readership. As written, I was originally slightly uncomfortable with the authors' framing of their work as being in contrast to "limited" previous discussions ("previous work was too specific, and this calls for the investigation of whether there exist any general principles ruling the interplay of randomness and structure"), because their introduction only referenced a small subset of the relevant literature.

This is not at all a criticism of the study (in fact, I think this entire broader literature would benefit from the authors' contribution here), merely a suggestion to perhaps slightly expand the framing/contextualization in the introduction:

- perhaps replacing "only equilibrium stability has received attention" by "equilibrium stability received particular attention, but…"
- And perhaps by adding a few more sentences and references out of the names above, plus Allesina (e.g. Allesina et al 2015 https://doi.org/10.1038/ncomms8842), O'Dwyer; maybe Crocker et al (biorxiv 635766)? -- whoever the authors feel is most relevant, to help orient the reader in the broader context (both within and beyond gLV).

You are absolutely correct, what we actually meant was that equilibrium stability is the prediction that has been tackled in the most systematic and general way, asking broadly and with similar Random Matrix Theory approaches how various types of structure can affect it, while most of the works going beyond linear dynamics have needed to choose somewhat particular combinations of structure and disorder (e.g. a bipartite interaction network for pure consumer-resource models), and focus on different subsets of predictions among a much broader range of questions – including more “functional” questions that may appear to only make sense in the context of a particular structure.

Of course we are not claiming to have the one right model that encompasses everything, nor the tool to solve it all, but rather that we focus on extracting conclusions that we have reason to believe will generalize the most widely, though counter-examples (both mathematically interesting and ecologically meaningful) are very much expected to occur.

We have now revised this part of the introduction to the following, in order to incorporate some suggested references and others, and better explain how these various studies differ (in their questions, in their methods, or in their results) and what has been done more or less systematically.

> *Our goal here is to investigate how predictions of fully random models are altered when species interactions possess a 'macroscopic', community-level structure. This question has motivated a vast array of theoretical studies, yet it is difficult to extract a general perspective from the literature, because of the sheer diversity of possible structures and the lack of a unified methodology and set of predictions to compare. Indeed, every possible structure potentially opens up new questions, such as asking about resource use efficiency and sequence in random consumer-resource models [14, 15], or trophic coherence in more complex food webs [16].*
>
> *Among possible questions, (linear) equilibrium stability has been studied most systematically: a range of studies have extended May's original approach, using developments in Random Matrix Theory to combine randomness with various specific structures, e.g. bipartite or trophic networks, spatial clusters, sparse interactions or complex correlations [17, 18, 19]. Departures from this linear approach have gone in very diverse directions, and while the popularization of some techniques (e.g. Dynamical Mean Field Theory, presented below) has focused efforts on a more unified subset of predictions, studies have still found disparate consequences to the addition of structure: causing stability to increase, decrease or both [20, 21]; affecting coexistence positively or negatively [22, 3, 23]; inducing distinct dynamical phases [24, 25]. We thus ask whether there could exist any broad principles in the interplay of randomness and structure, which, while not capturing the full range of these explorations, can provide baseline expectations on what features of random models are broken or conserved when interactions are partially structured [26].*

We hope that this helps clarify in which ways our work is novel and relevant and in which ways it draws from the existing literature.

Minor issues:

Line 57: citing reference 9 here is consistent with the purported conclusion of that paper, but the authors may wish to consult https://doi.org/10.1101/2024.03.26.586891 which casts doubt whether that example does in fact belong in this list.

Thank you for this reference, we had been looking for this kind of investigation given the lack of good null models for some of these claims, and we now mention this caveat in our introduction.

Would "sensitivity" be a better term in this context than "sensibility"?

• Sensitivity: the capacity of an organism or organ to sense and respond to stimulation
• Sensibility: the capacity to perceive or feel, particularly in relation to emotions, aesthetic appreciation, or moral values; sensitivity.

Thanks, that was a mistaken calque from our native Romance languages.

Lines 170-171: I'm not sure I understood this sentence. We are meant to conclude that in this regime, everything is straightforward, but what are these ODEs that collective quantities satisfy? Eq. (5) expresses species dynamics in terms of collective quantities, but the latter are in turn composed of species dynamics. Am I missing something obvious?

The claim of the sentence is in fact only correct for group structures (in which case the ODE is the low-dimensional Lotka-Volterra with interaction matrix given by the block structure of μ). For more complicated structures one can only claim that the collective variables are functions of one another, but one cannot exhibit a simple ODE. We have removed the sentence.

Reviewer 3

The manuscript "Structured and random interactions interplay in complex ecosystems dynamics" presents a theoretical study of ecological community dynamics, focusing on a hybrid interaction matrix that combines structured and random components. Leveraging tools from the statistical physics of disordered systems, the authors construct a framework that interpolates between fully structured and fully random generalized Lotka–Volterra (gLVE) models. Within this framework, they show that the contributions of structure and randomness can be disentangled, yet their interplay can lead to nontrivial dynamical outcomes.

I appreciate the approach and the theoretical effort to move beyond oversimplified gLVE models. While many of the resulting phenomena are not entirely unexpected, I believe the main value of the work lies in the formalism it develops, which provides a first step towards an unified and flexible perspective to study complex ecosystems.

Thank you very much for your kind and thoughtful words. We indeed hoped for our approach to help establish a baseline of rather general (if not absolutely universal and exception-free) expectations, which might be shared across a variety of more specific applications. The framework should help pinpointing what may be more or less surprising in each of them, and how even some apparently novel phenomena may in fact result from the same basic features interacting in slightly different ways. All reviewers have pointed to some failings in the presentation, which we hope to have addressed now, so that the precise scope of what we claim to be true and what we claim to be novel is clearer.

That said, there are some conceptual limitations that should be addressed to avoid overstating the novelty of the results and to better clarify the implications of the study. In particular, my main concern relates to the novelty of the findings. The phase diagram of gLVE models is well understood in terms of the first two moments of the interaction matrix (mean and variance of A_{ij}) and their correlations (i.e., $cov(A_{ij}, A_{ji})$). The structured component introduced here via the matrix μ_{ij} can, in principle, affect all three. What is not entirely clear to me is

whether the proposed formalism reveals genuinely new dynamical regimes beyond what can be expected from modified first and second moments, or whether it simply offers a nontrivial but ultimately reducible way to modulate these quantities. In the latter case, the methodological contribution would remain valuable, but the novelty of the results should be downscaled accordingly. If, however, new behaviors emerge that are not captured by moment-based predictions alone, this would constitute a significant theoretical advance.

We address this point in detail below, but this comment has helped us significantly refine our message. There is a key difference between the overall A_ij and the “structured component” mu_ij, namely, that the latter is exactly or effectively low-rank, either by definition or due to our choice of scaling (see reply to Comment 1 below).
What this means is that the universality or "concentration" properties that apply to a purely random A_ij (in the spirit of the Central Limit Theorem, i.e. all details of underlying parameters cease to matter except for mean and variance) do not apply to mu_ij.
When looking at mu_ij and its associated functional variables, its `macroscopic’ details really matter. One technical incarnation of that difference is that mu_ij contributes somewhat “volatile” outlier eigenvalues whereas the random component contributes a much more robust bulk. The two components contribute to determining both abundance distributions and stability.
Thus, adding mu_ij is not just effectively changing the moments of A_ij. Even if mu_ij is also drawn at random, it will be a small set of random numbers, and thus every draw can be very different from the others (e.g. some causing cycles while others cause bistability …) whereas the aggregate properties of every large draw become very similar (allowing universal dynamical phases and so on).
Our section 3.3 somewhat blurs the limits between small and large numbers since there are many groups, yet only a few survive at high abundance at any given point.

We have extensively revised the section on the definition of the interaction matrix, and added two new schematic Figures 1 and 2 in order to clarify the relative roles of its structured and disordered components.

To help strengthen the manuscript and clarify its contributions, I outline below several major points that I believe should be addressed before the paper can be considered for publication.

Comments

1. Early in the manuscript, the authors introduce a community of S interacting species structured by n_F community-level functions. Implicit in the formulation is the assumption that $n_F \ll S$, which is necessary to ensure that the structural part of the interaction matrix remains low-rank and analytically tractable compared to the disordered component. I suggest the authors make this assumption explicit.

   In fact, this can be viewed as an implicit assumption or as a consequence of our scaling choice: for the structured interaction matrix to have an overall impact that is comparable to that of the random one (or of intra-specific competition, i.e. being of order 1 in our dimensionless equation), it can only have a few components that are not negligibly

small. Thus, even if we assumed a model that in principle could have very large n_F, e.g. a full phylogenetic tree with every possible subtree being a function, in fact only a subset of size << S would matter. This is now mentioned in the discussion of Eq (4), and detailed in SI.

2. Moreover, it appears that each species can, in principle, contribute to or be affected by multiple functions, which could lead to a combinatorial explosion in the number of possible trait combinations. If this interpretation is correct, the requirement that $n_F \ll S$ becomes even more stringent to maintain model simplicity and interpretability. While the manuscript focuses primarily on cases where species belong to clearly separable functional groups, the general formalism allows for mixed affiliations. It would be useful to clarify whether such mixed cases are within scope and, if not, to state this explicitly.

   In our model, n_F is the number of functions in the community, not the number of ways to combine them. For instance, if each function is a resource and there are ten resources, each species will indeed have $2^10=1024$ ways of allocating which resources it consumes. Yet, one will still have $n_F=10$ and the rank of the matrix will be small. Similarly, if the traits represent group affiliations, allowing species to belong to different groups increases the number of combinations, but doesn't change n_F, which is just the number of groups.

   In fact, whether groups/resources etc. are disjoint or not can be determined by looking at whether the trait vectors $\mathcal{I},\mathcal{S}$ have disjoint support or not. But the number of functions is the same in both cases. Mixed cases are thus within scope, as the revised Figure 1 hopefully clarifies.

3. I appreciated the use of the I and S notation to define, respectively, the impact and sensibility traits of species in relation to community-level functions. This formulation offers a clean and ecologically meaningful way to encode structure into the interaction matrix. However, I found the current presentation—particularly the use of Figures 1 and 2—to be somewhat repetitive and fragmented. It may be more effective to provide a unified and concise introduction early on that explains the possible ecological interpretations of these traits, referencing the two modeling scenarios illustrated in Figures 1a and 1b as examples (e.g., functional groups, consumer-resource relationships).
   Additionally, the redefinition of the model in Section 3.3 using strains nested within a set of n_F species risks creating confusion, as it may suggest to the reader that this constitutes a distinct framework. In reality, it is a straightforward application of the same coarse-graining principle—just at a different level of biological organization. I suggest clarifying this explicitly and framing it from the very beginning just as an example of hierarchical coarse-graining (e.g., species into functional groups, strains into species), rather than reintroducing the model from scratch.

We agree with these comments and have significantly modified the figures in ways which we hope address all these concerns, and avoided the model redefinition in Section 3.3.

4. Sections 3.1 and 3.2 develop the theoretical framework under the implicit assumption that the system reaches a stationary state. While this assumption is explicitly acknowledged only later in Section 3.3, I believe it should be clearly stated upfront, as it underpins the subsequent derivations. Moreover, it would be helpful if the authors discussed the range of validity of this assumption more transparently. In particular, do the results shown in Figures 4c and 4d (which track functional magnitude and species richness as functions of σ) continue to hold if σ increases further? Does the system eventually leave the fixed-point regime and enter chaotic dynamics as predicted by the theory in Section 3.3? If so, I suggest to explicitly state that and to use word "intermediate" rather than "large" σ. I believe including a brief comment or additional data point would help contextualize the applicability of the results and avoid giving the impression that the equilibrium-based analysis is universally valid.

Section 3.1 is not limited to equilibrium see e.g. Roy et al 2019 for Dynamical Mean Field Theory in nonequilibrium but time-translation invariant states (chaotic fluctuations in their case). We now however clarify the limitations of Section 3.2 upfront.

5. In Eq. (9), the authors define the community-level correlation as the equal-time correlator $C^\star = \lim_{t \to \infty} C(t, t) = \lim_{t \to \infty} \langle x_i(t)^2 \rangle$, which corresponds to the variance of species abundances at stationarity. However, given that the underlying DMFT formalism involves stochastic processes with memory (colored noise), one might naturally expect a role for two-time correlations $C(t, t - \tau$. I suggest to clarify this choice.

Two-time correlations of the form $C(t,s)$ indeed play a role in the dynamics of the model. In particular, in the fixed point phase, they play a role in the transient. For long-term dynamics, however, the fixed point implies that $\lim_t x_i(t) = \lim_t x_i(t-tau)$ and therefore $\lim_t C(t,t) = \lim_t C(t,t-tau)$ for any τ, which is why $C^\star$ captures the properties of the noise at the fixed point. Another way to see it is that, since the $x_i(t)$ converge, the noises $\xi_i(t)$ converge as well to some-time independent noises $\xi^\star_i$. These are characterized by their variance which, by continuity, must equal $\lim_t C(t,t)$.

6. In lines 223–226 and Figure 5a, the authors show that a power-law-shaped species abundance distribution (SAD) can emerge when the total abundance is the only functional variable and the sensibility traits S_i^λ are drawn from a power-law distribution. While this reproduces an SAD form often observed in ecological datasets, I find the example potentially misleading in the context of the paper.
As far as I understand, drawing traits from a power-law in this way implicitly raises the rank of the structural matrix μ_{ij}, bringing it closer to a high-dimensional or even

full-rank object. This seems to contradict the premise that the structured component should be low-rank, which is central to the analytical tractability of the model. In this sense, the example becomes analogous to using non-Gaussian noise in the interaction matrix—something already explored, for instance, in recent work by Azaele & Maritan (PRL, 2024), where higher-order cumulants play a role.
Moreover, this effect occurs at $\sigma = 0$, i.e. in the fully structured regime. This makes it feel somewhat artificial or ad hoc, and not representative of the interplay between structure and randomness that is the core contribution of the paper.

The referee is right in saying that the power law is not a result of the interplay, but a purely structural feature. Our goal was to show that the SAD is very fragile to changes in the structure and that observed patterns might not be the result of universality in the random model. However, we agree that this is misleading, so we have removed the sentence; and moved the Figure to supplementary materials as an illustration.

Still, in line with previous comments, we wish to clarify the fact that power-law traits do not raise the rank of the structural matrix. In fact, in the example of the main text, it has rank 1.

7. As previously mentioned, I find the digression on plankton ecology at the beginning of Section 3.3 unnecessary for the core theoretical argument. I would suggest simply stating that the analysis refers to a coarse-graining level where each "species" aggregates a number of "strains", i.e., a specific instance of n_F functional units.

We now avoid this digression.

8. I found the dynamical behavior shown in Figure 6 particularly intriguing and worthy of further discussion. It appears that the dynamics at the strain and species levels remain qualitatively similar across the parameter sweep in σ: the two levels seem to share the same dynamical phase (e.g., chaotic, stationary), although the variability at the strain level is consistently higher. What I find most surprising—and potentially insightful—is the non-monotonic behavior of the system as σ increases. First, I was not expecting the system to display chaos at $\sigma=0$. If I understand correctly, even when the disordered component is formally zero, the system may exhibit chaotic dynamics if the structured component μ_{ij} has sufficient variance. In other words, the effective variance of the full interaction matrix is

$$
\sigma_{\text{eff}}^2 = a_1 \text{Var}(\mu_{ij}) + a_2 \sigma^2,
$$

(where the coefficients depend on S and n_F) which may be non-zero even when $\sigma = 0$, leading to a non-trivial position in the phase diagram of the corresponding random model. Is this interpretation correct?

The situation described in that Figure is a bit complex: while chaos appears both at low

and high sigma, it is not the same chaos, in several meaningful ways.
(1) The chaos that arises at sigma=0, has been described in Mallmin et al. 2024 for random, “strong” (that do not scale with S) interactions between macroscopic groups (formerly ‘species’). In this case, only a few groups dominate at any given time, and the rest have extremely low abundances. The ‘collective’ chaos observed for standard GLVs, where a large fraction of species have non negligible abundances, is retrieved for weak interaction strength.
(2) the group-level chaos at sigma=0 can also be observed at the species level, it “cascades down” the scales, whereas the high-sigma species-level chaos barely “trickles up” (in the large S limit, there would be no trace of it at all in macroscopic observables such as average species abundance). To put it differently, at low heterogeneity single-species chaotic trajectories are synchronized , hence fluctuations of the macroscopic observables like group abundances are preserved in the large S limit. For large σ, on the other hand, trajectories are desynchronized and the group averages undergo only finite-size fluctuations around zero.

What this means is that, rather than a non-monotonic phenomenon that combines the macroscopic variance of groups with the microscopic variance of species, we have two distinct phenomena, originating at two different scales, and they clash with each other. These two phenomena also originate in different parts of the spectrum of the interaction matrix and Jacobian – one is tied to outlier eigenvalues due to the relatively low-rank structured part (even though we are blurring the lines between low and high dimensions in this specific example, per (1) above there are in fact only a few really important groups at any given instant), the other is due to a continuous bulk of eigenvalues that cross the instability threshold and thus create a continuum of unstable dynamical modes.

We find this example particularly interesting because it means that one can put complexity at either the microscopic or the macroscopic scale (in the “randomness” or in the “structure”), and observe stability at either the microscopic or macroscopic scale, and the conclusions will not be the same. Unfortunately, that also makes for a somewhat challenging story to convey.

We therefore refer to another paper where we delve in the mathematical details of the two alternative routes (collective phase transition vs low-codimensional bifurcation) that are generically observed when microscopic heterogeneity is added to structured dynamical systems in out-of-equilibrium regimes.

We have modified the paragraph reporting this behaviour and the corresponding figure (now Figure 5) so as to point out the synchronous and asynchronous nature of the two chaotic regimes.

9. Furthermore, the non-monotonic behavior observed as σ increases (e.g., the transition from chaos to stability and back to chaos) suggests that adding structured components can impact not just the variance of interactions, but also their correlation

structure. This would imply that the system is not simply moving along a one-dimensional axis in a known gLVE phase diagram, but is instead exploring a more complex region of parameter space involving the correlations. Given that these effects directly touch on the core message of the paper—how structure and randomness interact—I would strongly encourage the authors to investigate this behavior more systematically, or at least to provide a more detailed theoretical interpretation of the observed transitions.

Hopefully the replies above and improvements to the text are sufficient to clarify that the non-monotonic behavior is due to two different mechanisms (one having to do with the bulk, the other with outliers of the spectrum), rather than feeding into a single mechanism in a complex way (e.g reshaping the bulk via correlations)
