# Supplementary material for: Interplay of structured and random interactions in complex ecosystems dynamics
Source: PLoS Comput Biol. 2025 Dec 26;21(12):e1013786. doi: 10.1371/journal.pcbi.1013786 (PMC12755798; doi:10.1371/journal.pcbi.1013786)
Supplement: Attachment — Submitted filename: response.pdf [file pcbi.1013786.s005.pdf]

Response to second referral for "Interplay of structured and random interactions in complex ecosystem dynamics"

Juan Giral Martínez, Matthieu Barbier, Silvia De Monte

Dear Editor,

We are very grateful for the opportunity to perform a second round of corrections to our manuscript, and for the time taken by the referees and yourself to help us improve its quality. We are glad that the referees consider out first round of modifications to have improved the article, and we wish to thank you and Referee 2 for your careful reading of the manuscript and for your additional recommendations, which have helped us polish the remaining rough edges.

You will find below a point-by-point answer to all your queries. We have also attached a version comparison between this revision and the previous one.

As per the journal requirements, we have removed the duplicated Supplementary Figures. They are now included in the Supplementary Information file only.

Sincerely yours,

Juan Giral Martínez, Matthieu Barbier & Silvia De Monte

1 Additional Editor Comments :

I concur with all three reviewers that this is a substantially improved presentation of your work. While Reviewers 1 and 3 are satisfied with the current version, Reviewer 2 and I have some minor comments, particularly regarding the figures, which may help improve the manuscript further.

We thank you very much for your additional comments and careful reading.

L23. This sentence is ambiguous. Are the patterns less robust to breaking equivalence than they are to the qualitative dynamics, or are the patterns less robust to breaking equivalence than the qualitative dynamics are?

We have rephrased the sentence in order to remove the ambiguity: *“ We conclude that whether interaction structure matters depends on the pattern: when breaking species equivalence, static patterns of species presence and abundance predicted from random interaction models are less robust than the qualitative nature of dynamical regimes.”*

L141. ”Impact traits” should be ”sensitivity traits”.

We have corrected this mistake.

L203. cancel out (no hyphen).

We have corrected the typo.

Fig 1. The matrix examples remain unclear. For instance, which aspect of matrix B tells us that there are two public goods here (water and pH)? In matrix C, which square size corresponds to which taxonomic unit (species, genus, family)? Matrix A should be especially clear since functional groups are the focal structure in the study. It can improve if the reader can connect the colors of the squares, which represent interactions between the functional groups, to the width of the arrows in the diagram. I suggest making all arrow widths visibly different and using a color gradient for the squares that makes their magnitude clear without needing a legend (which is understandably omitted).

The point is about the connection between the diagrams and the matrices. We have made it more explicit by adding details to the caption. For panel A, we have added “so that the block structure of the matrix reflects the group structure of the community”. For panel B, “in which case the rank of the matrix is bounded by the number of public goods”. For panel C, “as encoded by the fractal structure of the interactions (larger blocks correspond to higher-order taxa)”. We hope that these make the Figure clear for all readers.

Fig 2. Neither the caption nor axis labels fully explain what is being plotted in the equilibrium plots. It looks like those are species abundance distributions, with abundance on the x-axis and number of species on the y-axis. But this should be explicitly mentioned (for example, should be defined, or better still, replaced with words).

We have followed your recommendation and that of Reviewer #2 and relabeled the axes to “equilibrium abundances” and “SAD” (the acronym is defined in the caption). We have also labeled all other axes in the Figure.

In the caption to Fig 3 and elsewhere in the text, it is repeatedly stated that when the magnitude of random interactions is set to zero, the blue group (group 1) is extinct. However, this model includes immigration. To avoid con-

fusion, the language should be changed to something along the lines of "group 1 subsists at background levels, rescued from extinction by immigration."

We have changed the wording. In order to avoid confusion with the "rescue" caused by randomness, we have preferred to use the word "excluded" when referring to species that remain at the migration threshold. We hope this makes the notions clearer.

L280 "past some critical value of " is a restrictive modifier, so it should not be set off with commas.

We have corrected this.

Fig. 5 caption: remove duplicated word "group" before "contains" on the third line.

We have corrected the typo.

L388: typo "As a consequence".

We have corrected the typo.

On a general note, I disagree with the implied interpretation of the term in Eq. (4) as standing in contrast with or independent from functional traits (e.g. line 409). Rather, it arguably stems from contributions to the interaction matrix from unobserved or unmodeled traits and/or functions. It is quite distinct from random demographic or environmental fluctuations, which can affect changes in dynamics and coexistence outcomes apart from the influence of species interactions. This could be easily addressed by a note early on clarifying that the random component of the interaction matrix reflects latent ecological structure, not extrinsic noise.

We completely agree with your interpretation. In order to avoid giving the impression of a contrast between functional traits and randomness, we have rephrased the appropriate sentences, in general by using the words "structural traits" instead of "functional traits". We have also added a note in line 119, when the matrix model is presented: "Other potential sources of stochasticity, such as demographic or environmental fluctuations, are not included in the model". We have also modified the labels of Figure 2.

Journal Requirements:

1) We note that your Supplementary Figures files are duplicated on your submission as they are uploaded separately and included in the supporting information file. Please remove any unnecessary or old files from your revision, and make sure that only those relevant to the current version of the manuscript

are included.

Note: Supplementary figures should be uploaded with the file type 'Supporting Information' rather than 'Figure'. Please ensure that each Supporting Information file has a legend listed in the manuscript after the references list.

2) We have noticed that you have uploaded Supporting Information files, but you have not included a list of legends. Please add a full list of legends for your Supporting Information files after the references list.

Note: If the reviewer comments include a recommendation to cite specific previously published works, please review and evaluate these publications to determine whether they are relevant and should be cited. There is no requirement to cite these works unless the editor has indicated otherwise.

2 Reviewers' comments

2.1 Reviewer #1

The authors have increased the quality of the presentation of their results. I believe that the current version of the manuscript is suitable for publication.

We sincerely thank Reviewer #1 for their time and careful reading of the manuscript and the appendices. Their comments have allowed us to greatly improve the quality and readability of the article, and we are thankful for considering that is now ready for publication.

2.2 Reviewer #2

I thank the authors for making the extensive revisions. I find the clarity of the manuscript to be greatly improved; which I hope will help the manuscript achieve the impact it deserves. I also appreciated the reorganizing of some material in to the SI vs main text. Unfortunately, the scale of changes may have created a couple rough edges that could use some smoothing (e.g. a notation not defined until later, or an abrupt / seemingly unmotivated narrative transition.). I mention these minor issues below.

We wish to thank Reviewer #2 for their time and careful reading of the manuscript. We have addressed all the additional points, which have allowed us to polish such last rough edges. We are also thankful for their encouraging comments since our initial submission.

My one larger new comment is about Figure 5. I find the reorganized version to be rather unsatisfying. Previously, there was a clear punchline: a non-monotonic behavior, and the illustrative panels were followed by a summary panel delivering this message. Based on the response to the referees, the authors now feel uncomfortable calling out this ”nonmonotonicity”, since (as they explain in the response to reviewer 3) ”it is not the same chaos”, but actually two separate effects.

To be honest, I would see no harm in calling this ”nonmonotonic” – the variance goes down and then goes back up. The authors understand this well, to the point that they can explain that the origin of the chaos in the two cases is different. This seems like a point that should make the narrative stronger, not weaker: “look, the variance behaves non-monotonically, and we understand exactly where this is coming from!”

But the authors’ chosen solution took all the punch out and made this figure completely ”toothless”. Although the summary panel has been dropped, the rest of the figure presentation remains extremely suggestive of non-monotonicity (the arrow of sigma connecting the panels; chaos, then no chaos, the chaos again...). But now this suggestiveness never goes anywhere. Merely showing a few panels with *examples* of dynamics with no systematic quantification seems rather weak. A reader skimming the figures (before reading the text) is not presented with any coherent summary or punchline for this figure, meaning they are invited to draw some conclusion themselves from the examples shown (never a good idea). But worse, the most natural conclusion is conspicuously – and (for a reader who did NOT read the response to reviewers) inexplicably! – avoided, and the text appears to dance around this point (e.g. the word ”non-monotonic” has been scrubbed from both legend and text). To me it seems that the desire to be extra precise turned an interesting point into something much weaker and confusing.

Following the referee’s recommendation we have again included the bottom graph with the fluctuation scale at the two levels of description. We have restored the references to the non-monotonic effect of heterogeneity on microscopic stability both in the caption and in the text, while keeping the difference between the micro and the macro notions of stability as a key point of the argument.

Minor points:

- **Figure 1B**, bottom - while other bottom-row illustrations are self-explanatory, for this one it might be helpful to clarify in the legend that this illustration is meant to illustrate a low-rank structure (induced by this competition/influence of public goods).

Following your recommendation and that of the Editor, we have added a clarification of the link between public goods and matrix rank in the caption: "**B.** the availability or magnitude f_λ of a small set of public goods (e.g. resources or environmental variables), in which case the rank of the matrix is bounded by the number of public goods".

- **Figure 2** legend never defines x*. In fact, this figure is first referenced in line 197, but x* isn't defined until line 237 (which is actually full two pages later – please note that line numbering in LaTeX is incompatible with some equation environments, and half of your lines aren't numbered...)

  Following your recommendation and that of the Editor, we have avoided using this notation in the Figure, and replaced it by "equilibrium abundances".

- **In Figure 4**, the graph of how $f_1^\star$ and $f_2^\star$ interact is not very clear (and has no panel label). Also, the legend should specify what lines vs dots mean.

  We have added a label to the first panel of the figure, and we have added two symbols indicating that the interaction between the groups is competitive. We hope that this clarifies the figure.

- **Figure 4B** - I'd suggest explicitly reminding the reader what quantity is denoted "phi" denotes (would be especially appreciated by reader scanning figures before reading the paper text).

  We have added ϕ to the caption so as to remind the reader of its meaning.

- **Figure 4C**: The claim of non-monotonicity in "both groups" is not exactly spectacular. For both blue and gray lines it hinges on a single (last) point. Would this be supported more strongly if you included a few more points on the large-sigma end?

  We have added a few points between $\sigma = 1.4$ and $\sigma = 1.6$ in order to show that ϕ keeps decreasing beyond the maximum.

- **Unnumbered line following line 323**: "We now turn to a theoretical explanation of the two possible ways species can transition out of the intermediate-randomness equilibrium": I found this transition a little jarring; the flow could be improved.

  We have reworked the transition. It now reads *"We now turn to a theoretical explanation of the dynamical regimes highlighted in Figure 5. We focus on the stability of the intermediate-randomness equilibrium, and show*

how the transition towards synchronized out-of-equilibrium dynamics (for small σ) and that towards microscopic chaotic dynamics (for large σ) can be related, respectively, to the random and the structured part of the interactions.”.

2.3 Reviewer #3

I sincerely thank the authors for their efforts in revising the manuscript, which, in my opinion, is now suitable for publication.

We are very grateful to Reviewer #3 for their time and for their careful reading of our manuscript. As a result, our article is now more understandable and of higher quality. We thank them for considering that it is now worthy of publication.
