# Supplementary material for: Interplay of structured and random interactions in complex ecosystems dynamics
Source: PLoS Comput Biol. 2025 Dec 26;21(12):e1013786. doi: 10.1371/journal.pcbi.1013786 (PMC12755798; doi:10.1371/journal.pcbi.1013786)
Supplement: Attachment — Submitted filename: Review.docx [file pcbi.1013786.s003.docx]

The manuscript “Structured and random interactions interplay in complex ecosystems dynamics” presents a theoretical study of ecological community dynamics, focusing on a hybrid interaction matrix that combines structured and random components. Leveraging tools from the statistical physics of disordered systems, the authors construct a framework that interpolates between fully structured and fully random generalized Lotka–Volterra (gLVE) models. Within this framework, they show that the contributions of structure and randomness can be disentangled, yet their interplay can lead to nontrivial dynamical outcomes.

I appreciate the approach and the theoretical effort to move beyond oversimplified gLVE models. While many of the resulting phenomena are not entirely unexpected, I believe the main value of the work lies in the formalism it develops, which provides a first step towards an unified and flexible perspective to study complex ecosystems.

That said, there are some conceptual limitations that should be addressed to avoid overstating the novelty of the results and to better clarify the implications of the study. In particular, my main concern relates to the novelty of the findings. The phase diagram of gLVE models is well understood in terms of the first two moments of the interaction matrix (mean and variance of $A_{ij}$) and their correlations (i.e., $cov(A_{ij}, A_{ji})$). The structured component introduced here via the matrix $\mu_{ij}$ can, in principle, affect all three. What is not entirely clear to me is whether the proposed formalism reveals genuinely new dynamical regimes beyond what can be expected from modified first and second moments, or whether it simply offers a nontrivial but ultimately reducible way to modulate these quantities. In the latter case, the methodological contribution would remain valuable, but the novelty of the results should be downscaled accordingly. If, however, new behaviors emerge that are not captured by moment-based predictions alone, this would constitute a significant theoretical advance.

To help strengthen the manuscript and clarify its contributions, I outline below several major points that I believe should be addressed before the paper can be considered for publication.

**Comments**

1. Early in the manuscript, the authors introduce a community of $S$ interacting species structured by $n_F$ community-level functions. Implicit in the formulation is the assumption that $n_F \ll S$, which is necessary to ensure that the structural part of the interaction matrix remains low-rank and analytically tractable compared to the disordered component. I suggest the authors make this assumption explicit.
   Moreover, it appears that each species can, in principle, contribute to or be affected by multiple functions, which could lead to a combinatorial explosion in the number of possible trait combinations. If this interpretation is correct, the requirement that $n_F \ll S$ becomes even more stringent to maintain model simplicity and interpretability. While the manuscript focuses primarily on cases where species belong to clearly separable functional groups, the general formalism allows for mixed affiliations. It would be useful to clarify whether such mixed cases are within scope and, if not, to state this explicitly.
2. I appreciated the use of the $I$ and $S$ notation to define, respectively, the impact and sensibility traits of species in relation to community-level functions. This formulation offers a clean and ecologically meaningful way to encode structure into the interaction matrix. However, I found the current presentation—particularly the use of Figures 1 and 2—to be somewhat repetitive and fragmented. It may be more effective to provide a unified and concise introduction early on that explains the possible ecological interpretations of these traits, referencing the two modeling scenarios illustrated in Figures 1a and 1b as examples (e.g., functional groups, consumer-resource relationships).
   Additionally, the redefinition of the model in Section 3.3 using strains nested within a set of n_F species risks creating confusion, as it may suggest to the reader that this constitutes a distinct framework. In reality, it is a straightforward application of the same coarse-graining principle—just at a different level of biological organization. I suggest clarifying this explicitly and framing it from the very beginning just as an example of hierarchical coarse-graining (e.g., species into functional groups, strains into species), rather than reintroducing the model from scratch.
3. Sections 3.1 and 3.2 develop the theoretical framework under the implicit assumption that the system reaches a stationary state. While this assumption is explicitly acknowledged only later in Section 3.3, I believe it should be clearly stated upfront, as it underpins the subsequent derivations. Moreover, it would be helpful if the authors discussed the range of validity of this assumption more transparently. In particular, do the results shown in Figures 4c and 4d (which track functional magnitude and species richness as functions of $\sigma$) continue to hold if $\sigma$ increases further? Does the system eventually leave the fixed-point regime and enter chaotic dynamics as predicted by the theory in Section 3.3? If so, I suggest to explicitly state that and to use word "intermediate" rather than "large" $\sigma$. I believe including a brief comment or additional data point would help contextualize the applicability of the results and avoid giving the impression that the equilibrium-based analysis is universally valid.
4. In Eq. (9), the authors define the community-level correlation as the equal-time correlator $C^\star = \lim_{t \to \infty} C(t, t) = \lim_{t \to \infty} \langle x_i(t)^2 \rangle$, which corresponds to the variance of species abundances at stationarity. However, given that the underlying DMFT formalism involves stochastic processes with memory (colored noise), one might naturally expect a role for two-time correlations $C(t, t - \tau$. I suggest to clarify this choice.
5. In lines 223–226 and Figure 5a, the authors show that a power-law-shaped species abundance distribution (SAD) can emerge when the total abundance is the only functional variable and the sensibility traits S_i^\lambda are drawn from a power-law distribution. While this reproduces an SAD form often observed in ecological datasets, I find the example potentially misleading in the context of the paper.
   As far as I understand, drawing traits from a power-law in this way implicitly raises the rank of the structural matrix $\mu_{ij}$, bringing it closer to a high-dimensional or even full-rank object. This seems to contradict the premise that the structured component should be low-rank, which is central to the analytical tractability of the model. In this sense, the example becomes analogous to using non-Gaussian noise in the interaction matrix—something already explored, for instance, in recent work by Azaele & Maritan (PRL, 2024), where higher-order cumulants play a role.
   Moreover, this effect occurs at $\sigma = 0$, i.e. in the fully structured regime. This makes it feel somewhat artificial or ad hoc, and not representative of the interplay between structure and randomness that is the core contribution of the paper.
6. As previously mentioned, I find the digression on plankton ecology at the beginning of Section 3.3 unnecessary for the core theoretical argument. I would suggest simply stating that the analysis refers to a coarse-graining level where each “species” aggregates a number of “strains”, i.e., a specific instance of $n_F$ functional units.
7. I found the dynamical behavior shown in Figure 6 particularly intriguing and worthy of further discussion. It appears that the dynamics at the strain and species levels remain qualitatively similar across the parameter sweep in $\sigma$: the two levels seem to share the same dynamical phase (e.g., chaotic, stationary), although the variability at the strain level is consistently higher. What I find most surprising—and potentially insightful—is the non-monotonic behavior of the system as $\sigma$ increases. First, I was not expecting the system to display chaos at $\sigma=0$. If I understand correctly, even when the disordered component is formally zero, the system may exhibit chaotic dynamics if the structured component $\mu_{ij}$ has sufficient variance. In other words, the effective variance of the full interaction matrix is
   $$
   \sigma_{\text{eff}}^2 = a_1 \text{Var}(\mu_{ij}) + a_2 \sigma^2,
   $$
   (where the coefficients depend on $S$ and $n_F$) which may be non-zero even when $\sigma = 0$, leading to a non-trivial position in the phase diagram of the corresponding random model. Is this interpretation correct?
8. Furthermore, the non-monotonic behavior observed as $\sigma$ increases (e.g., the transition from chaos to stability and back to chaos) suggests that adding structured components can impact not just the variance of interactions, but also their correlation structure. This would imply that the system is not simply moving along a one-dimensional axis in a known gLVE phase diagram, but is instead exploring a more complex region of parameter space involving the correlations. Given that these effects directly touch on the core message of the paper—how structure and randomness interact—I would strongly encourage the authors to investigate this behavior more systematically, or at least to provide a more detailed theoretical interpretation of the observed transitions.
